# The University and Hospital Bulletin

**Published:** 1905-03

**Authors:** 


### The University and Hospital Bulletin

DEVOTED TO THE INTERESTS OF THE UNIVERSITY OF BUFFALO
AND THE HOSPITALS.

EDITED BY
NELSON W. WILSON, M. D.

*Assistants:*
E. R. McGuire, M. D., Burton T. Simpson, M. D.

Under the auspices of the Faculty of the Medical Department of the University

M. D. Mann, A. M., M. D., Dean,
Charles G. Stockton, M. D.,
John Parmenter, M. D.,
Eli H. Long, M. D.,
Roswell Park, A. M., M. D., LL. D.,
Charles Cary, M. D.,
Herbert M. Hill, A. M., Ph. D.,
Herbert U. Williams, M. D.

AND

*Buffalo General Hospital* Henry Reed Hopkins, M. D.
*Hospital of the Sisters of Charity* Eugene A. Smith, M. D.
*German Hospital* Herman E. Hayd, M. D.

### Teaching of Physiology—University of Buffalo.

IN RECENT years the methods of teaching physiology in medical schools have undergone considerable change. Didactic methods have, in some instances been entirely replaced by inductive methods. In other instances, laboratory work and practical demonstrations are being used, in part, as a substitute for, and, in part, as a supplement to lectures and quizzes. In some schools, notably the Harvard Medical School, the socalled concentration method is employed in teaching the preliminary branches of medicine. According to this plan, all the time of one semester is devoted to two or more closely allied subjects, such as anatomy, histology, embryology, and the like, or physiology and physiologic chemistry. These subjects are completed in one semester.

This plan of concentration has much to recommend it from the standpoint of pedagogy. The student devotes his entire time to one subject without having his attention distracted by other work. He is figuratively immersed in that particular branch and when he is taken out some of it is bound to stick. The instructors are worked hard during this period but they have the rest of the school year free for original investigation, a certain amount of which is now deemed necessary for every first-class institution. It has been found impracticable, however, to adopt this method in its entirety for every medical school. To carry it out, logically, requires a large and expensive equipment and a large corps of paid instructors. Different combinations of didactic and laboratory teaching, likewise, are used by different schools and teachers. It is probably true that no single method of instruction is applicable to all schools. Each teacher has to determine for himself what method or combination of methods gives him the best results.

In the medical department of the University of Buffalo, the outline of teaching in physiology is as follows: the subject is taught in the first two years of the medical course. The first year's work consists of lectures, recitations and practical demonstrations, four hours per week during both semesters being thus utilised. During this time the following subdivisions of physiology are thoroughly covered, chemical composition of the body, blood, circulation of the blood and lymph, applied physics, respiration, secretion and digestion. The work in anatomy and histology is correlated with this work.

The lecture, quiz and demonstration work is continued through the second year and includes, a review of secretion and digestion, absorption, metabolism, animal heat, excretion, general sensation, special senses, central nervous system, muscle nerve physiology, and a series of lectures on applied physiology. In this year a modification of the concentration method is employed by the departments of anatomy and physiology, the practical work in anatomy being largely confined to the first semester and that in physiology to the second semester.

The laboratory work, then, is begun and completed in the second semester of the sophomore year. Six hours per week for each student is devoted to this work. This is confined largely to the physical side of physiology, the chemical side being studied in the laboratory of chemistry. The ground covered is the following: first, muscle nerve physiology which is probably the most thoroughly worked out branch and most exact in results, although possibly, least applicable to practical medicine. It is chosen first, however, because, as above mentioned, exact results are more readily obtained, small animals, such as the frog may be used, material for experimentation is easy to get and keep. It serves also as an excellent training for the student in manual dexterity and careful observation and prepares him for more important work later.

This work includes, determination of the irritability of muscle and nerve to various stimuli, elasticity of muscle, work done by contracting muscle; speed of transmission of impulses over nerves; character of voluntary muscle contraction; influence of temperature and of fatigue upon muscle twitch; volume of contracting muscle; electric phenomena of muscle and nerve; electrotonus or the influence of the direct current upon the irritability and conductivity of nerve and muscle fibers; the reaction of degeneration and the like.

Following this, certain experiments on the physiology of the central nervous system are performed, such as are simple and for which the frog may be used. These consist of a study of reflex action; diffusion of impulses within the spinal cord; inhibition of reflexes; effect of drugs on reflex action; reaction time. Still using the frog, the study of the circulation of the blood is begun. This consists of the following experiments: graphic record of heart beat; effect of temperature; cardio-inhibition and acceleration; effect of stimulating vagus terminals; reflex cardio-inhibition; effects of drugs.

The study of the circulation is now continued on mammals, using the rabbit for this purpose. It is hardly necessary to state that all animals are completely narcotised with morphine and ether and are killed at the end of the experiment before consciousness has returned, so that no pain is inflicted.

The function of the cardiac nerves is thus studied; blood pressure and the action of drugs. The heart-beat, pulse and blood pressure in man, are also studied.

Under respiration, the respiratory capacity, mechanics of respiration and normal respiratory sounds in man are studied. In the rabbit, the action of the respiratory muscles and the nervous control of respiration are determined. The movements of swallowing, innervation and movements of stomach and intestines are likewise studied experimentally. In addition to this work, all of which is done by the students themselves, the following demonstrations are given by instructors to small divisions of the class: action of secretory nerves, mechanism of pancreatic secretion, perfusion of frog's and mammalian heart, circulation time, ablation of cerebrum, cerebellum, stimulation of motor cortical areas, hemisection of spinal cord, artificial eye, study of optics, ophthalmoscope, general sensation and special senses.

For laboratory work, the class is divided into two sections, each section spending two sessions, of three hours each per week, in the laboratory. The students work in groups of three or four, each group being supplied with complete equipment of apparatus. The students are obliged to do the work themselves, the function of the instructor being to supervise and suggest. In this way considerable knowledge is obtained first hand, and more important still, excellent training in manipulation and observation.

Pharmacology, under the direction of Dr. Kiepe, is taught in connection with the physiology. Much of the success of the work is due to the voluntary assistance of the corps of instructors and assistants, including Doctors VanBergen, Kiepe, Leonard, Mann, LeBreton and Plummer. The expense of running a successful laboratory course is considerable. It is our intention from time to time as finances permit, to increase the number of apparatus sets and decrease the number of students at a desk.

F. C. Busch, B. S., M. D.

---

### HERNIA OF THE BLADDER COMPLICATING INGUINAL HERNIA.

Not so very many years ago this condition was considered among the rarities in surgical practice and remained so classified until a comparatively recent period when careful investigation of the literature showed it to be really more frequent than was generally supposed. Coley, for instance, in a work edited by him in which 950 cases of hernia are reported, does not mention a single case of bladder complication. Later, however, he mentions two cases. Other writers, among them, Shepard, of Montreal, claim the bladder complication occurs in 1 per cent. of all cases.

Nevertheless, it is sufficiently rare as a complication to warrant the reporting of all cases, and the importance of the complication is so fully recognised as to make it almost obligatory on writers on genitourinary matters to refer to it as a possibility in discussing bladder conditions.

The cases which follow were operated in Dr. Park's service at the Buffalo General Hospital: the first was a man of 50, with a direct inguinal hernia. The sac was dissected out and a fluctuating mass was discovered beneath its site. Hernia of the bladder was suspected and the diagnosis was confirmed by the introduction of a catheter into the bladder through the urethra when the mass disappeared on the withdrawal of urine. The hernia was closed in the usual way.

The second case was also one of direct inguinal hernia, the patient a man of 36. The sac was small and just below it was felt a protrusion, which, on further examination, was found to be a soft mass. Catheter diagnosis was made as in the first case, followed by the complete disappearance of the tumor. The hernia was closed with reindeer tendon.

The importance of making accurate diagnosis in such cases is apparent. Usually, the bladder hernia is mistaken for cyst of the cord, and unless the operator has the possibility of a bladder condition in mind in all hernia operations he is quite likely to open the supposed cyst and only discover his mistake when there is a gush of urine which fills the wound,—at best, a most unfortunate and distressing accident.

In not all cases, however, is the protrusion of the bladder so marked as to be felt as a fluctuating tumor beneath the sac. In this event it may be several days before there is any ground for suspicion that all is not right. Then there appears bloody urine and abdominal distension, and the clinical picture of the true condition is revealed. The trouble in such cases is the accidental puncture of the bladder with the needle used in placing sutures to close the hernial opening.

The question of bladder protrusion complicating hernia has been very carefully investigated by Drs. Curtis and Brunner.

Curtis in the *Annals of Surgery*, Vol. xxi., 1895, collected forty-five cases which showed a mortality of 25 per cent. Brunner has collected 180 cases, all that have been reported for the last 100 years. In the *Deutsch Zeit. Chir.*, Band 47, 1898, he considers the matter in all its phases and discusses it in a most elaborate and entertaining manner.

---

### AN INTERESTING CASE OF MULTIPLE HERNIA.

An interesting and rare case of multiple hernia was operated during Dr. Hayd's service at the German Hospital. An Italian of 56 was admitted with a large femoral hernia on the right side, and right and left oblique complete inguinal hernias. The femoral hernia was first operated. The sac was opened, transfixed at the neck, tied off and removed. The ring was closed by purse string sutures. The right inguinal canal was opened and a Bassini radical operation was done. The sac was opened at the neck, the finger introduced and it was carefully separated from the canal. As the under surface of the sac was shelled out, the stump of the femoral hernia was pulled up into the wound. The operation was completed on this side and a Bassini radical closure done on the right.

A double inguinal and a femoral at the same time is a rare condition, very few such cases being reported. The patient was kept in bed three weeks and made an uninterrupted recovery.

---

### AN OBSCURE AND COMPLICATED APPENDIX CASE.

A woman of 45 years was sent to the German Hospital by Dr. Dorr with a history of distress in the right side covering a period of several months. The distress became acute pain two days prior to admission and the day before she came to the hospital Dr. Dorr had been called. On admission her temperature was 102½° ; pulse, 110. There was a large and tender mass at the junction of the upper and lower right abdominal quadrants, its longest diameter on a line with the umbilicus. A diagnosis of appendicitis was made and the woman was at once prepared for operation.

On opening the cavity the mass was found to be grayish-red in color and was apparently continuous with the liver. After careful manipulation a line of separation was discovered between the liver and the mass which was separated and found to be a huge organised blood clot in the web of the omentum, the latter being twice twisted on itself at this point. The mass was tied off and removed. The transverse colon appeared in the wound. It was followed to the right and the cecum located. From the outer, posterior surface of the cecum the appendix was found, very long and densely adherent to the liver. It was removed. Its coats were much thickened but there was little acute involvement. A small gauze drain was put into the wound to rest upon a suspicious looking spot on the hepatic flexure of the transverse colon. On the seventh day the drain was removed and for a few days a foul discharge persisted. It, however, ceased and the patient was discharged cured.

---

A CASE OF HYDATIFORM MOLE.

A Polish woman was admitted to Dr. Hayd's service at the German Hospital with this history: age, 24; married four years; one child living, three years old; ten months ago gave birth to a child which lived but a few hours; menstruation appeared regularly for five months; for the past three months has flowed almost continuously.

Upon examination the uterus was found irregular in outline and had a "doughy" feel. The cervix was closed and not at all softened. The woman was tamponed and in twenty-four hours discharged a large hydatiform mole. There was no fetus although the amniotic sac was complete and could be perfectly demonstrated. The decidual site of the membranes was thickened and had evidently been formerly attached to the uterus.

